# Swooning and Syncope

**Published:** 1909-02-06

**Authors:** 


### Diseases of Children.

### SWOONING AND SYNCOPE.

Sudden syncope may occur as the termination of many diseases and is the result of complete and permanent cessation of the heart's action. In its milder form it is called "fainting" or "swooning," and is both temporary in duration and more gradual in development. It may occur at all ages. Even babies have fainting attacks which are extremely difficult of diagnosis. More often it occurs in children at about puberty. Thus it is especially apt to take place in school-children during early morning chapel. The ill-ventilated building, empty stomach, erect posture, and lack of mental stimulation are conjoint exciting causes of the attack. Many such children exhibit vaso-motor instability in other forms, notably as orthostatic albuminuria, and in some no doubt the fainting is primarily due to early morning masturbation. Swooning is not uncommon in girls about puberty and in many is of the nature of hysteria rather than true syncope.

A fainting attack is ushered in by a feeling of faintness, a sense of increasing feebleness, sometimes a sinking feeling in the epigastrium which is probably due to vaso-dilatation in the splanchnic area, dimness or loss of sight because of lack of blood supply to the retina, pallor, sighing respiration, and a sense of nausea. The heart beat may be very feeble or there may be undue consciousness of the heart's action. The radial pulse is almost imperceptible, increased or decreased in frequency. Consciousness is lost, more or less completely, for a variable time, but not so abruptly as in epilepsy. The child sinks down or can lie down and rarely falls with sufficient suddenness to cause injury. Gradually consciousness and colour return, without mental confusion or special tendency to sleep. A very severe attack may be attended by muscular spasms.

A study of the symptoms shows that fainting is commonly due to alterations in intracranial pressure. Such alterations may arise from very simple causes: *e.g.* the sudden change from the recumbent to the erect posture; the prolonged maintenance of the erect posture when enfeebled, due to action of gravity; the rapid withdrawal of fluid from the circulation, as in loss of blood, profuse purging, too rapid evacuation of ascitic fluid, etc.; sudden changes in the circulation, the result of vaso-motor action, as on exposure to heat after the superficial vessels have been constricted by exposure to cold. Of nervous causes one must mention the sight and smell of blood, various odours to which some individuals seem peculiarly susceptible, sudden emotions of alarm or pleasure, and severe pain.

The mode of production is not quite clear, except in so far as the loss of consciousness is associated with cardiac failure. The point to be settled is whether this failure is the primary cause, producing faintness through the deficient supply of blood to the brain, or is primarily of cerebral origin. An actual fall in blood pressure, whether cause or sequel, is the chief determining factor. The fall in pressure is the consequence of inhibition of the heart and vaso-motor centre, and of dilatation in the splanchnic area. There is no doubt that the sudden cessation of the blood supply to the brain and consequent sudden fall in intracranial pressure may result in immediate death, as in some cases of aortic regurgitation. Similarly in a milder degree the rapid dilatation of the vessels in the skin or in the splanchnic area causes a rapid reduction in the blood supply to the brain and faintness. Thus it is clear that many cases are due to a cardio-vascular derangement causing diminution in the cerebral blood supply with the sequence of symptoms: a feeling of faintness or feebleness, pallor, and recovery or loss of consciousness. In other instances this effect is brought about by stimulation of the cardio-inhibitory centre in the medulla, reflexly, by severe pain, unpleasant sights, various odours and even sounds; or by direct transmission of the stimulus from the cortex of the brain to the centre in the bulb, as in severe emotion.

Applying these principles to the cases sometimes seen in babies we can diagnose attacks as the result of severe pain, such as colic; of various blood states, such as lymphatism; of various reflex stimuli; or due to epilepsy. It is most important and extremely difficult to differentiate *petit mal* from attacks of faintness, and indeed the less serious diagnosis is often made out of kindness to the parents though the more serious one is borne in mind. In *petit mal* the pallor of faintness is generally absent as an initial symptom. Loss of consciousness is sudden and recovery may be as sudden. Frequently there is some mental confusion on recovery, or the child may present hysteroid symptoms or pass into a sound sleep. Perhaps there is a family history of epilepsy or allied affections. Often there is some definite exciting cause of the attack, if one of swooning. Possibly both are due to the same cause, namely failure of the cerebral circulation, but differ in the conditions of their production and the tendency to recurrence. More probably epilepsy is the result of some nutritional change in the nerve cells, rendering them unduly susceptible to alterations in cerebral pressure and other stimuli. Intense cerebral congestion, such as may be induced by the paroxysms of whooping cough, may cause sudden syncope.

The treatment of fainting is quite simple. Either the posture of complete recumbency should be adopted with the head lowered, or the sitting posture with the head bent down low between the knees. The latter posture will often cause the feeling of faintness to pass off. The former should be adopted if swooning has occurred. Fresh air and the loosening of all constricting clothes about the neck and waist are essential. Smelling salts, tickling the nose with a feather, and stimulants are useful, but rarely necessary, for most attacks pass off quickly with recumbency and fresh air. A cold douche is a valuable means of stimulation and inducing vaso-constriction. Subsequently the cause should be ascertained and suitable treatment adopted.

